# Predictive value of the geriatric nutrition risk index for postoperative delirium in elderly patients undergoing cardiac surgery

**DOI:** 10.1111/cns.14343

**Published:** 2023-07-05

**Authors:** Zhiqiang Chen, Quanshui Hao, Rao Sun, Yanjing Zhang, Hui Fu, Shile Liu, Chenglei Luo, Hanwen Chen, Yiwen Zhang

**Affiliations:** ^1^ Department of Anesthesiology, Shunde Hospital Southern Medical University (The First People's Hospital of Shunde) Foshan China; ^2^ Department of Anesthesiology Huanggang Central Hospital of Yangtze University Huanggang China; ^3^ Department of Anesthesiology, Tongji Hospital, Tongji Medical College Huazhong University of Science and Technology Wuhan China

**Keywords:** cardiac surgery, elderly patients, geriatric nutrition risk index, postoperative delirium, risk factor

## Abstract

**Aims:**

The aims of the study were to determine the relationship between preoperative geriatric nutritional risk index (GNRI) and the occurrence of postoperative delirium (POD) in elderly patients after cardiac surgery and to evaluate the additive value of GNRI for predicting POD.

**Methods:**

The data were extracted from the Multiparameter Intelligent Monitoring in Intensive Care (MIMIC‐IV) database. Patients who underwent cardiac surgery and were aged 65 or older were included. The relationship between preoperative GNRI and POD was investigated using logistic regression. We determined the added predictive value of preoperative GNRI for POD by measuring the changes in the area under the receiver operating characteristic curve (AUC) and calculating the net reclassification improvement (NRI) and integrated discrimination improvement (IDI).

**Results:**

A total of 4286 patients were included in the study, and 659 (16.1%) developed POD. Patients with POD had significantly lower GNRI scores than patients without POD (median 111.1 vs. 113.4, *p* < 0.001). Malnourished patients (GNRI ≤ 98) had a significantly higher risk of POD (odds ratio, 1.83, 90% CI, 1.42–2.34, *p* < 0.001) than those without malnutrition (GNRI > 98). This correlation remains after adjusting for confounding variables. The addition of GNRI to the multivariable models slightly but not significantly increases the AUCs (all *p* > 0.05). Incorporating GNRI increases NRIs in some models and IDIs in all models (all *p* < 0.05).

**Conclusions:**

Our results showed a negative association between preoperative GNRI and POD in elderly patients undergoing cardiac surgery. The addition of GNRI to POD prediction models may improve their predictive accuracy. However, these findings were based on a single‐center cohort and will need to be validated in future studies involving multiple centers.

## Graphical abstract

We found a nonlinear relationship between postoperative delirium risk and preoperative GNRI scores. The risk of delirium decreased sharply until the GNRI reached approximately 126, after which it tended to rise gradually. Adding GNRI to delirium prediction models may improve their accuracy.

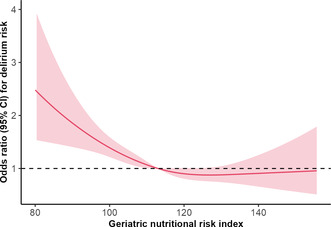

## 1 INTRODUCTION

Delirium is an acute brain disorder characterized by fluctuating changes in awareness, attention, perception, and cognition.^1^ There is a high incidence of delirium among patients undergoing cardiac surgery,^2^, ^3^ with the elderly having the greatest risk.^4^ Postoperative delirium (POD) has been shown to prolong hospital stays, negatively affect patient quality of life, increase economic burdens, and cause long‐term cognitive impairments.^5^, ^6^, ^7^, ^8^ There are preventive measures that can lower the incidence of POD.^9^, ^10^, ^11^, ^12^, ^13^ Therefore, it is crucial to identify patients at risk for delirium as early as possible and implement these preventative measures.

Some studies have suggested that malnutrition may be a risk factor for POD, but the evidence is inconclusive.^14^, ^15^, ^16^, ^17^ Malnutrition is common among the elderly, especially those hospitalized or with a chronic illness.^18^, ^19^ In surgical patients, poor nutritional status is associated with a number of adverse postoperative outcomes, including infectious complications, impaired wound healing, and delayed recovery.^20^, ^21^, ^22^ Recent studies also found a correlation between malnutrition and POD in patients undergoing coronary artery bypass grafting (CABG).^23^, ^24^ Nevertheless, their small sample sizes (one with 99 patients and the other with 398 patients) prevented them from conducting a multivariate analysis adjusting all variables or reaching a definitive conclusion. In these studies, malnutrition was evaluated using questionnaire‐based tools. However, the use of these tools in the elderly population may be limited due to recall bias and communication difficulties, which may lead to inaccurate assessments. Several objective indices, including the geriatric nutritional risk index (GNRI),^25^ have been developed to evaluate nutritional status. As a simple and objective index, the GNRI overcomes the limitation of questionnaire‐based tools, as it allows clinicians to evaluate the nutritional status of patients solely based on their height, weight, and serum albumin level.

In elderly surgical patients, GNRI has been shown to be predictive of adverse postoperative outcomes, such as infectious complications, poor rehabilitation, and prolonged hospital stays.^26^, ^27^, ^28^ However, the relationship between GNRI and POD has rarely been studied,^29^ particularly in patients undergoing cardiac surgery. In the present study, we conducted a retrospective study to investigate whether preoperative GNRI score was associated with the development of POD in elderly patients undergoing cardiac surgery. This study utilized data from the Multiparameter Intelligent Monitoring in Intensive Care (MIMIC‐IV) database. The MIMIC‐IV is an open‐access database containing comprehensive clinical data of patients admitted to intensive care units (ICUs) or emergency departments.^30^ The database also includes a substantial number of cardiac surgery patients. Using these data, we also evaluated the additive value of GNRI in the prediction of delirium after cardiac surgery.

## 2 MATERIALS AND METHODS

### 2.1 Data source

The study data were extracted from a publicly accessible database, the MIMIC‐IV (version: v1.0).^30^ The database contains comprehensive clinical data for ICU patients at Beth Israel Deaconess Medical Center from 2008 to 2019. One author completed the online training for the Collaborative Institutional Training Initiative program of the National Institutes of Health and was approved by the Institutional Review Boards of the Massachusetts Institute of Technology to access the database and acquire the data of MIMIC‐IV. The reporting of this study complies with the transparent reporting of a multivariable prediction model for individual prognosis or diagnosis (TRIPOD) statement.^31^

### 2.2 Patient selection

Patients were included if they met the following criteria: (1) aged ≥65 years; (2) underwent cardiac surgery such as CABG, valvular surgery, aortic aneurysm surgery, or a combination thereof; (3) were admitted to the ICU after surgery; (4) had body weight, height, and serum albumin data (within 30 days before surgery) for calculation of GNRI. Table S1 provides a list of the International Classification of Diseases (ICD)‐9 and ICD‐10 codes that are used to identify cardiac surgeries. We excluded patients who (1) underwent other surgeries, (2) had a history of schizophrenia, or (3) had dementia. In the case of patients who had undergone more than one cardiac surgery, only the first one was considered.

### 2.3 Data extraction

The data were extracted from the MIMIC‐IV database using structured query language with PostgreSQL. The following information was extracted: (1) demographic information: age, sex, ethnicity, weight, and height; (2) comorbidities: hypertension, myocardial infarction, congestive heart failure, peripheral vascular disease, cerebrovascular disease, obstructive sleep apnea, diabetes, chronic pulmonary disease, chronic liver disease, and chronic renal disease; (3) lifestyle factor: alcohol abuse; (4) type of surgery: CABG, valve surgery, aortic replacement, as well as a combined cardiac surgery; (5) laboratory findings before surgery and on the first day in the ICU: peripheral white blood cell count, hemoglobin, serum albumin, and serum creatinine; (6) mean values of vital signs on the first day in the ICU: heart rate, mean arterial blood pressure (MAP), percutaneous oxygen saturation (SpO_2_), and respiratory rate; (7) the Sequential Organ Failure Assessment (SOFA) score on the first day in the ICU; (8) use of benzodiazepines during ICU stay; (9) outcomes: delirium during hospital stay, ICU and hospital stay lengths, and hospital mortality. The data extraction code is accessible on GitHub (https://github.com/MIT‐LCP/mimic‐iv). The ICD‐9 and ICD‐10 codes used to identify cardiac surgeries and delirium are presented in Table S1.

For patients included in the MIMIC‐IV database, delirium was screened through the confusion assessment method for the intensive care unit (CAM‐ICU) and diagnosed with the Diagnostic and Statistical Manuals of Mental Disorders (DSM‐5).^32^ The GNRI was calculated using the following formula^25^, ^33^: (1.489 × 10 × serum albumin (g/dL)) + (41.7 × weight (kg)/ideal body weight (kg)). The ideal body weight was calculated as follows^25^: 0.75 × height (cm)—62.5 for male patients, 0.60 × height (cm)—40 for female patients. In addition, we divided the included patients into two groups: those with no risk of malnutrition (GNRI > 98) and those at risk of malnutrition (GNRI ≤ 98).^25^

### 2.4 Statistical analysis

The data distribution of continuous variables was evaluated using the Shapiro–Wilk test, and the results showed they were all non‐normal. Therefore, continuous variables were expressed as median (interquartile range [IQR]) and compared with the Mann–Whitney *U*‐test or Wilcoxon rank sum test. The categorical variables were expressed as counts (percentages) and compared using the χ^2^ test or Fisher's exact test, as appropriate.

Logistic regression was used to investigate the association between preoperative GNRI (as both a continuous and a categorical variable) and POD, and the odds ratio (OR) with 95% confidential interval (CI) was calculated. First, we conducted a univariate analysis, and then we gradually added other variables to conduct multivariate analyses: model 1: adjusting demographic information, comorbidities, and lifestyle factors; model 2: adjusting the variables in model 1 as well as preoperative laboratory findings; model 3: adjusting the variables in model 2 in addition to the type of surgery; model 4: adjusting the variables in model 3 as well as postoperative factors (i.e., the laboratory findings, vital signs, and SOFA score on the first day in the ICU, as well as the use of benzodiazepines during ICU stay). To avoid multicollinearity, albumin and body mass index (BMI) were not included in these models. Additionally, we employed backward stepwise logistic regression (model 5) to obtain an optimal prediction model with the lowest Akaike information criterion (AIC).^34^ Collinearity between continuous variables was evaluated using the variance inflation factor (VIF) and variables with a VIF greater than five were omitted from the final model.^35^ Moreover, we fitted restricted cubic splines to visualize the association between preoperative GNRI and POD.

We calculated the sensitivity and specificity of GNRI for predicting POD. Moreover, we also assessed the added predictive value of preoperative GNRI for POD using the following methods.^36^ First, we quantified the predictive ability of each model using the area under the receiver operating characteristic curve (AUC) and then used DeLong's method to determine the change in AUCs after the addition of GNRI. Second, we calculated the indices of net reclassification improvement (NRI) and integrated discrimination improvement (IDI) to evaluate the risk reclassification capability of GNRI. Finally, we conducted a χ^2^ likelihood ratio test to determine if the model including GNRI provided a more accurate fit than the model without it.

All analyses were conducted using R version 4.2.1 (http://www.R‐project.org, The R Foundation). In our study, a two‐tailed *p*‐value of <0.05 was considered statistically significant.

## 3 RESULTS

### 3.1 Clinical characteristics

**FIGURE 1:**
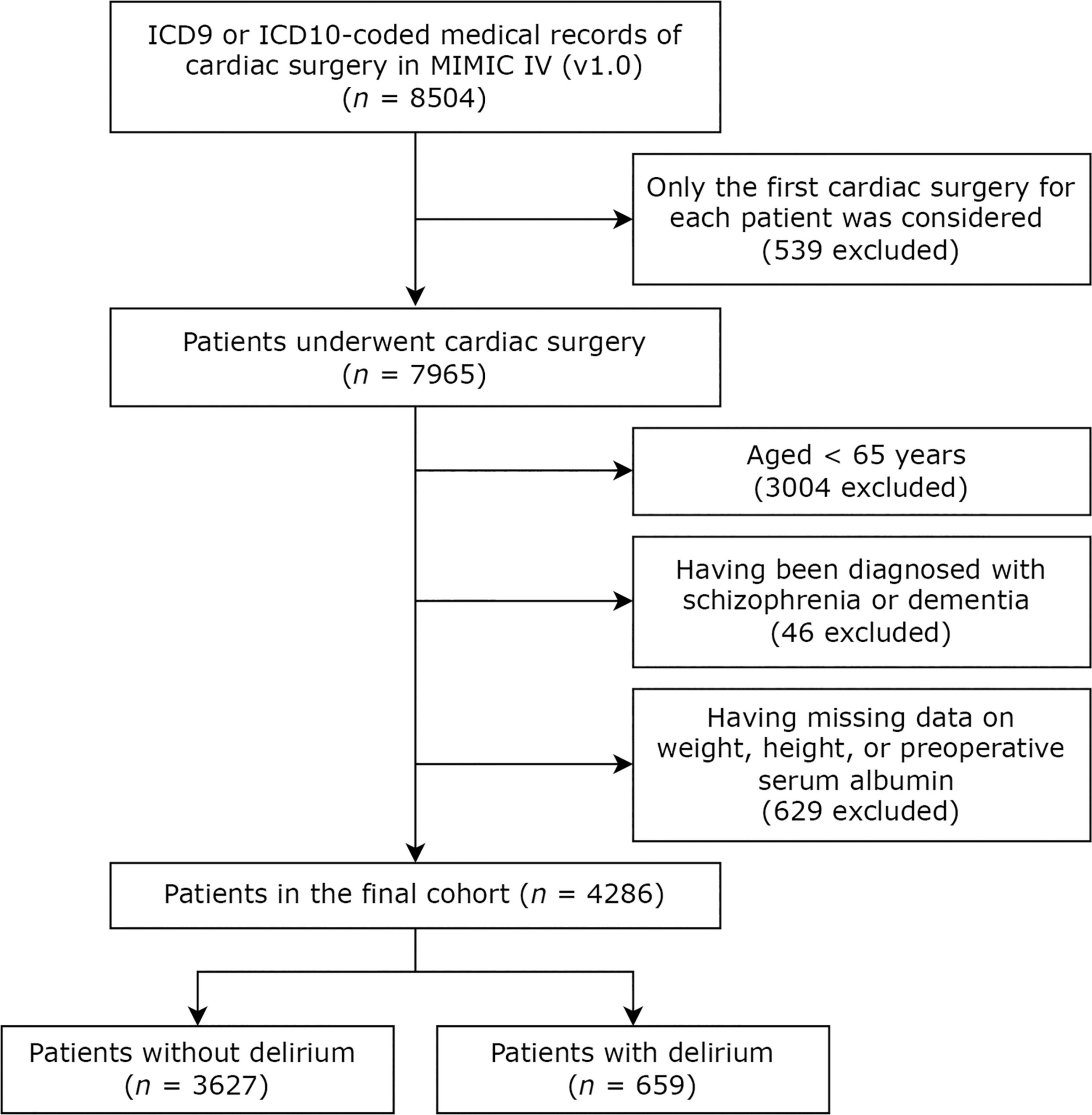
Flow diagram for patient selection.

A total of 4286 patients were included for analysis. Figure 1 shows the selection process for patients. The median age of the included patients was 74 years (IQR: 70–79 years), and 31.1% were female. 2254 patients (52.6%) underwent CABG surgery, 1023 patients (23.9%) underwent valve surgery, 53 patients (1.2%) underwent aortic replacement surgery, and 956 patients (22.3%) underwent combined cardiac surgery. The median length of ICU and hospital stays were 2 days (IQR 1–3 days) and 7 days (IQR 5–11 days), respectively. 659 patients (16.1%) developed delirium after surgery, and 108 patients (1.8%) died in the hospital. Table 1 shows the characteristics of the study cohort, both globally and stratified by the presence of POD. The patients with delirium were more likely to be older, to have a lower BMI, to have a history of alcohol abuse, and to have more comorbidities. These patients were also more likely to utilize benzodiazepines following surgery and had higher SOFA scores on their first day in the ICU. Patients who experienced POD had lower preoperative GNRI scores than those who did not (median 111.1 vs. 113.4, *p* < 0.001, Table 1). Patients who underwent valve surgery had a higher preoperative GNRI compared with those who underwent CABG (median 114.3 vs. 112.9, *p* = 0.003) and those who underwent combined cardiac surgery (median 114.3 vs. 112.4, *p* = 0.008, Table S2).

**TABLE 1:** Characteristics of the included patients.

	Total, *n* = 4286	No delirium, *n* = 3627	Delirium, *n* = 659	*p*‐value
Age, years	74 (70, 79)	74 (69, 79)	77 (72, 82)	<0.001
Female sex	1333 (31.1%)	1119 (30.9%)	214 (32.5%)	0.435
Ethnicity				
White	3287 (76.7%)	2787 (76.8%)	500 (75.9%)	0.624
Others	999 (23.3%)	840 (23.2%)	159 (24.1%)	
BMI, kg/m^2^	27.9 (24.8, 31.5)	28.0 (24.9, 31.5)	27.5 (24.1, 31.2)	0.006
Comorbidity				
Hypertension	3551 (82.9%)	2989 (82.4%)	562 (85.3%)	0.081
Myocardial infarction	1247 (29.1%)	1031 (28.4%)	216 (32.8%)	0.027
Congestive heart failure	1230 (28.7%)	964 (26.6%)	266 (40.4%)	<0.001
Peripheral vascular disease	681 (15.9%)	519 (14.3%)	162 (24.6%)	<0.001
Cerebrovascular disease	494 (11.5%)	388 (10.7%)	106 (16.1%)	<0.001
Obstructive sleep apnea	494 (11.5%)	408 (11.2%)	86 (13.1%)	0.206
Diabetes	1574 (36.7%)	1314 (36.2%)	260 (39.5%)	0.124
Chronic pulmonary disease	980 (22.9%)	803 (22.1%)	177 (26.9%)	0.009
Chronic liver disease	134 (3.1%)	97 (2.7%)	37 (5.6%)	<0.001
Chronic kidney disease	841 (19.6%)	656 (18.1%)	185 (28.1%)	<0.001
Alcohol abuse	106 (2.5%)	72 (2.0%)	34 (5.2%)	<0.001
Preoperative laboratory findings				
Albumin, g/dL	4.0 (3.7, 4.3)	4.0 (3.8, 4.4)	4.0 (3.6, 4.3)	<0.001
Creatinine, mg/dL	1.0 (0.8, 1.2)	1.0 (0.8, 1.2)	1.0 (0.9, 1.3)	<0.001
Hemoglobin, g/dL	12.7 (11.2, 13.8)	12.7 (11.3, 13.9)	12.3 (10.9, 13.5)	<0.001
White blood cell counts, ×10^9^/L	7.2 (5.9, 8.7)	7.2 (5.9, 8.6)	7.5 (6.2, 9.0)	0.001
Preoperative GNRI	113.0 (105.3, 121.3)	113.4 (105.7, 121.5)	111.1 (102.6, 119.6)	<0.001
Malnutrition risk				
Without risk (>98)	3894 (90.9%)	3328 (91.8%)	566 (85.9%)	<0.001
With risk (≤98)	392 (9.2%)	299 (8.2%)	93 (14.1%)	
Type of surgery				
CABG	2254 (52.6%)	1964 (54.1%)	290 (44.0%)	<0.001
Valve surgery	1023 (23.9%)	887 (24.5%)	136 (20.6%)	
Aortic replacement	53 (1.2%)	35 (1.0%)	18 (2.7%)	
Combined cardiac surgery	956 (22.3%)	741 (20.4%)	215 (32.6%)	
Laboratory findings on the first day in ICU				
Creatinine, mg/dL	1.0 (0.8, 1.2)	0.9 (0.8, 1.2)	1.0 (0.8, 1.3)	<0.001
Hemoglobin, g/dL	8.7 (7.8, 9.8)	8.8 (7.9, 9.9)	8.4 (7.6, 9.4)	<0.001
White blood cell counts, ×10^9^/L	15.2 (12.1, 19.2)	15.2 (12.2, 19.1)	15.4 (11.8, 19.7)	0.570
Vital signs on the first day in ICU				
Heart rate, beats/min	80 (75, 86)	80 (75, 86)	80 (75, 86)	0.699
MAP, mmHg	73 (69, 76)	73 (70, 77)	72 (69, 76)	0.002
SpO_2_, %	98 (97, 99)	98 (97, 99)	98 (97, 99)	0.743
Respiration rate, breaths/min	17 (16, 19)	17 (16, 19)	17 (16, 19)	0.001
SOFA score on the first day in ICU	5 (4, 8)	5 (4, 7)	7 (5, 9)	<0.001
Use of benzodiazepines during ICU stay	1258 (29.4%)	948 (26.1%)	310 (47.0%)	<0.001
Length of ICU stay, days	2 (1, 3)	2 (1, 3)	3 (2, 6)	<0.001
Length of hospital stay, days	7 (5, 11)	7 (5, 10)	10 (7, 15)	<0.001
Hospital mortality	56 (1.3%)	35 (1.0%)	21 (3.2%)	<0.001

*Note*: Values are expressed as median (interquartile range) or number of patients (%).

Abbreviations: BMI, body mass index; CABG, coronary artery bypass grafting; GNRI, geriatric nutritional risk index; ICU, intensive care unit; MAP, mean arterial blood pressure; SOFA, Sequential Organ Failure Assessment; SpO_2_, percutaneous oxygen saturation.

In addition, we separated the included patients into two groups: those without risk of malnutrition (GNRI > 98) and those at risk of malnutrition (GNRI ≤ 98). As shown in Table S3, patients at risk for malnutrition typically had lower hemoglobin, creatinine, and BMI levels. They also tended to be older, female, and more likely to develop delirium after surgery.

### 3.2 The association between preoperative GNRI and POD

In the univariate logistic regression analysis, patients at risk of malnutrition (GNRI ≤ 98) had a significantly higher risk of POD (OR, 1.83, 90% CI, 1.42–2.34, *p* < 0.001) than those without malnutrition (GNRI > 98; Table 2 and Table S4). After adjusting for confounding factors in models 1, 2, 3, and 4, this relationship was attenuated. Nevertheless, GNRI ≤ 98 remained independently associated with a higher risk of POD (all *p* < 0.05; Table 2 and Table S4).

**TABLE 2:** Association between GNRI‐assessed malnutrition risk and delirium after cardiac surgery.

	Univariable analysis; crude OR (95% CI), *p*‐value	Multivariable analysis model 1; adjusted OR (95% CI), *p*‐value	Multivariable analysis model 2; adjusted OR (95% CI), *p*‐value	Multivariable analysis model 3; adjusted OR (95% CI), *p*‐value	Multivariable analysis model 4; adjusted OR (95% CI), *p*‐value
Without malnutrition risk (GNRI > 98)	Ref.	Ref.	Ref.	Ref.	Ref.
With malnutrition risk (GNRI ≤ 98)	1.83 (1.42–2.34), *p* < 0.001	1.47 (1.12–1.91), *p* < 0.005	1.51 (1.15–1.98), *p* < 0.003	1.56 (1.18–2.04), *p* < 0.001	1.61 (1.22–2.13), *p* < 0.001

Abbreviations: CI, confidential interval; GNRI, geriatric nutritional risk index; OR, odds ratio.

We used backward stepwise logistic regression to obtain a model with the smallest AIC value. The prediction model was completed by incorporating 13 variables, and the minimum AIC value was 3331. Among these variables, GNRI ≤ 98 (OR, 1.60, 95% CI, 1.22–2.09, *p* = 0.001), older age (OR, 1.07, 95% CI, 1.05–1.08, *p* < 0.001), a history of peripheral vascular disease (OR, 1.40, 95% CI, 1.11–1.75, *p* = 0.004), obstructive sleep apnea (OR, 1.38, 95% CI, 1.06–1.80, *p* = 0.017), diabetes (OR, 1.21, 95% CI, 1.01–1.46, *p* = 0.043), and alcohol abuse (OR, 2.48, 95% CI, 1.57–3.87, *p* < 0.001), aortic replacement surgery (OR, 2.38, 95% CI, 1.21–4.55, *p* = 0.010), a combined surgery (OR, 1.43, 95% CI, 1.15–1.77, *p* = 0.001), higher respiration rate (OR, 1.05, 95% CI, 1.02–1.09, *p* = 0.004), and higher SOFA score (OR, 1.10, 95% CI, 1.07–1.14, *p* = 0.017) on the first day in ICU, and benzodiazepines use during ICU stay (OR, 2.18, 95% CI, 1.81–2.62, *p* < 0.001) were independently associated with an increased risk for POD (Table 3).

**TABLE 3:** Variables selected by the stepwise logistic regression analysis.

	OR (95% CI)	*p*‐value
Age, years	1.07 (1.05, 1.08)	<0.001
Ethnicity		
Others	Ref.	
White	0.85 (0.69, 1.05)	0.130
Peripheral vascular disease		
No	Ref.	
Yes	1.40 (1.11, 1.75)	0.004
Cerebrovascular disease		
No	Ref.	
Yes	1.26 (0.98, 1.61)	0.072
Obstructive sleep apnea		
No	Ref.	
Yes	1.38 (1.06, 1.80)	0.017
Diabetes		
No	Ref.	
Yes	1.21 (1.01, 1.46)	0.043
Chronic liver disease		
No	Ref.	
Yes	1.39 (0.89, 2.11)	0.134
Alcohol abuse		
No	Ref.	
Yes	2.48 (1.57, 3.87)	<0.001
Malnutrition risk		
Without risk (GNRI > 98)	Ref.	
With risk (GNRI ≤ 98)	1.60 (1.22, 2.09)	0.001
Type of surgery		
CABG	Ref.	
Valve surgery	0.93 (0.74, 1.18)	0.564
Aortic replacement	2.38 (1.21, 4.55,)	0.010
Combined cardiac surgery	1.43 (1.15, 1.77)	0.001
Creatinine on the first day in ICU, mg/dL	1.10 (0.99, 1.22)	0.061
Mean respiration rate on the first day in ICU, breaths/min	1.05 (1.02, 1.09)	0.004
SOFA score the first day in ICU	1.10 (1.07, 1.14)	<0.001
Use of benzodiazepines during ICU stay		
No	Ref.	
Yes	2.18 (1.81, 2.62)	<0.001

Abbreviations: CABG, coronary artery bypass grafting; CI, confidential interval; GNRI, geriatric nutritional risk index; ICU, intensive care unit; OR, odds ratio; SOFA, Sequential Organ Failure Assessment.

**FIGURE 2:**
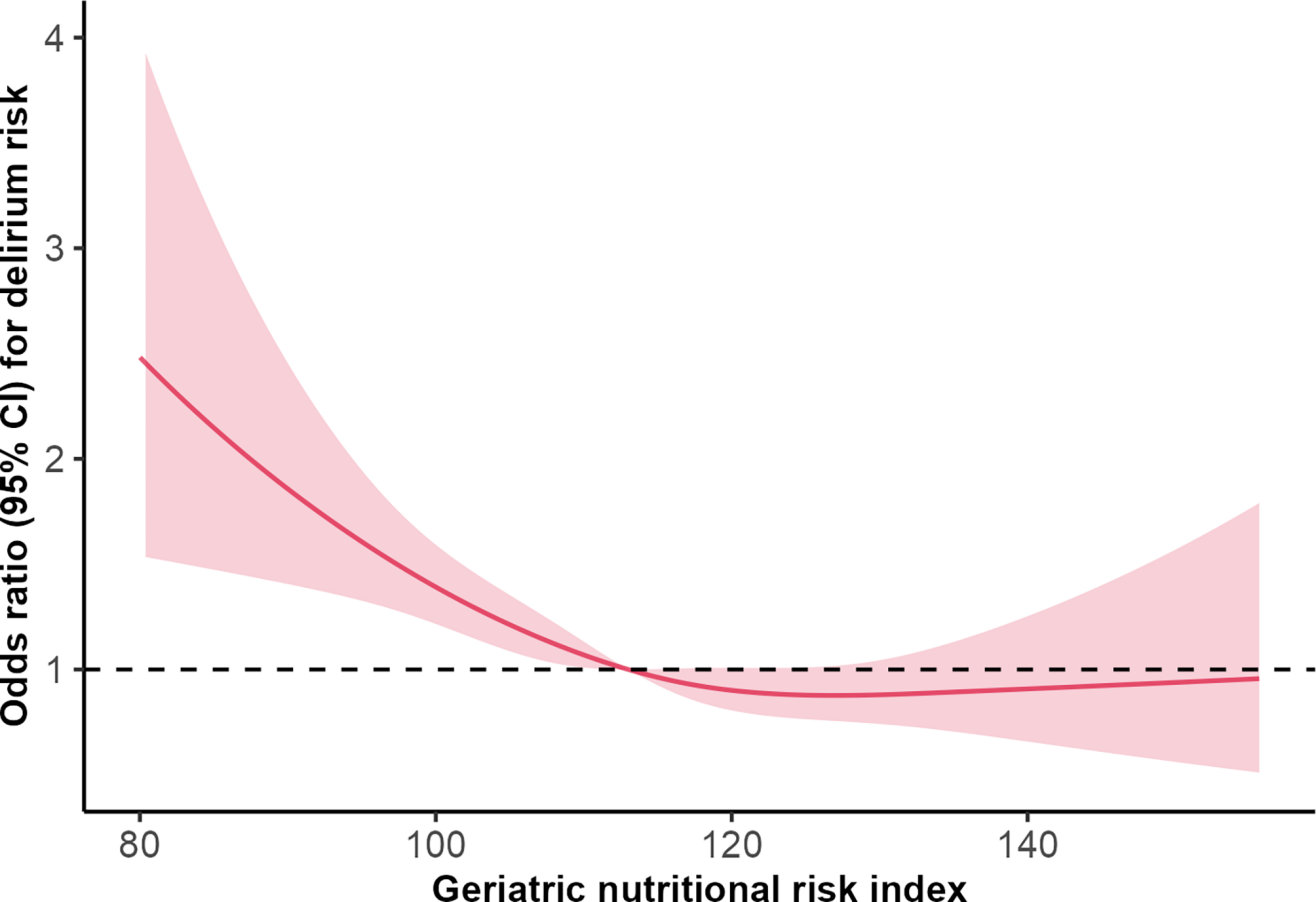
Restricted spline curves for the relationship between the geriatric nutritional risk index and delirium in elderly cardiac surgery patients. The red bold line denotes the odds ratio, while the shaded area represents the 95% confidence intervals.

We also treated GNRI as a continuous variable to investigate its association with POD. As shown in Table S5, after adjusting for confounding variables in models 1, 2, 3, and 4, a lower GNRI was independently associated with an increased risk for POD (all *p* < 0.05). We employed restricted cubic splines to illustrate the relationship between preoperative GNRI and POD. As shown in Figure 2, a nonlinear association was observed between the OR of POD and GNRI. The OR decreased sharply until the GNRI reached approximately 126, after which it tended to rise gradually.

### 3.3 Additive value of GNRI in delirium risk prediction

The sensitivity and specificity of GNRI for predicting POD were 0.14 and 0.92, respectively. The predictive power of each model was quantified by calculating AUCs. The addition of GNRI to the base models resulted in a slight increase in the AUCs, but the increase was not statistically significant (all *p* > 0.05; Table 4). NRIs and IDIs were calculated to evaluate the risk reclassification capability of the models. The incorporation of GNRI increases NRIs in model 2 (*p* = 0.020) and model 3 (*p* = 0.013) and IDIs in all five models (all *p* < 0.05; Table 4), suggesting that the incorporation of GNRI may help improve the predictive models' capacity for risk reclassification. In addition, we performed a likelihood ratio test. In models 1, 2, 3, 4, and 5, the addition of GNRI significantly improved the accuracy of delirium prediction (all *p* < 0.05; Table 4).

**TABLE 4:** Performance metrics of multivariable models with and without GNRI to predict delirium after cardiac surgery.

	AUC		Net reclassification improvement		Integrated discrimination improvement		Likelihood ratio test, *p*‐value
	Index (95% CI)	*p*‐value for Δ AUC	Index (95% CI)	*p*‐value	Index (95% CI)	*p*‐value	
Model 1 without GNRI	0.676 (0.654–0.697)						
Model 1 with GNRI	0.680 (0.658–0.701)	0.078	0.0295 (−0.036, 0.095)	0.374	0.0019 (0.0001, 0.0037)	0.042	0.006
Model 2 without GNRI	0.680 (0.659–0.702)						
Model 2 with GNRI	0.685 (0.663–0.701)	0.066	0.0240 (−0.045, 0.093)	0.493	0.0023 (0.0003, 0.0042)	0.020	0.004
Model 3 without GNRI	0.691 (0.669–0.712)						
Model 3 with GNRI	0.695 (0.673–0.716)	0.094	0.040 (−0.030, 0.110)	0.264	0.0026 (0.0005, 0.0047)	0.016	0.002
Model 4 without GNRI	0.730 (0.709–0.750)						
Model 4 with GNRI	0.734 (0.714–0.754)	0.052	0.050 (−0.021, 0.120)	0.166	0.0025 (0.0003, 0.0048)	0.027	0.001
Model 5 without GNRI	0.726 (0.705–0.746)						
Model 5 with GNRI	0.730 (0.710–0.750)	0.068	0.103 (0.040, 0.167)	0.001	0.0026 (0.0003, 0.0049)	0.026	<0.001

Abbreviations: AUC, area under the receiver operating characteristic curve; CABG, coronary artery bypass grafting; CI, confidential interval; GNRI, geriatric nutritional risk index; ICU, intensive care unit; OR, odds ratio; SOFA, Sequential Organ Failure Assessment.

## 4 DISCUSSION

In the present study, we retrospectively analyzed the data of 4286 elderly patients who had undergone cardiac surgery. Our results demonstrate that a lower preoperative GNRI score was independently associated with an increased risk of POD. Furthermore, we assessed the additive value of GNRI in predicting the risk of POD, and the results indicated that the incorporation of GNRI into POD prediction models improved their accuracy and risk reclassification capability.

Malnutrition is prevalent among elderly surgical patients and has been shown to be associated with a high risk of a variety of adverse outcomes, such as infectious complications, impaired wound healing, and a deterioration in recovery.^20^, ^21^, ^22^ Several studies have investigated the relationship between malnutrition and POD, primarily in patients undergoing noncardiac surgery.^14^, ^15^, ^16^, ^17^ The majority of these studies demonstrated that malnutrition is an independent POD risk factor.^14^, ^15^, ^17^ However, a recent study revealed contradictory findings.^16^ In their multivariate logistic analysis, Zhang et al.^16^ determined that malnutrition was not associated with POD. The inconsistent findings may be partly explained by the variability in the types of surgeries and malnutrition screening tools that they employed.

Recent studies also examined the relationship between preoperative nutritional status and POD in patients undergoing cardiac surgery.^23^, ^24^ A study conducted by Ringaitiene assessed the nutritional status of 99 patients undergoing on‐pump CABG by using the Nutritional Risk Score 2002 (NRS‐2002) before the surgery.^23^ They found that patients with POD were significantly more likely to suffer from malnutrition (NRS‐2002 score ≥ 3) than those without. Velayati et al.^24^ examined the data of 398 adult patients undergoing CABG surgery. According to their findings, malnutrition, as measured by the NRS‐2002 or Subjective Global Assessment, was associated with the occurrence of POD. Although these studies found a correlation between malnutrition and POD in cardiac surgical patients, their small sample size prevented them from adjusting for all confounding variables in a multivariate analysis or reaching a definitive conclusion. In the present study, we included a large sample size of elderly patients undergoing cardiac surgery (4286 patients), which allows us to adjust more variables (28 variables) to determine the predictive value of malnutrition for POD.

To date, several tools have been developed to assess the nutrition status of individuals.^19^, ^37^ The Mini Nutritional Assessment‐Short Form, the Malnutrition Universal Screening Tool, and the NRS‐2002 have been validated for diagnosing malnutrition and predicting clinical outcomes.^37^ However, due to recall bias and communication difficulties, these tools may not be as effective with the elderly and may result in inaccurate evaluations. By contrast, the GNRI is a simple and objective indicator that does not require patient cooperation and can be used in all clinical settings.^25^ It has been demonstrated that the GNRI can predict the prognosis of cancer patients, patients with heart failure, patients undergoing hemodialysis, and patients with stroke.^38^, ^39^, ^40^, ^41^, ^42^ Moreover, the GNRI has also been suggested as a prognostic indicator for surgical patients.^43^, ^44^, ^45^ In patients undergoing cardiac surgery, a lower GNRI was independently associated with delayed postoperative rehabilitation, longer ICU and hospital stays, and an increased mortality rate.^27^, ^28^, ^46^ However, previous research has not examined the relationship between GNRI and delirium after cardiac surgery. In the present study, a negative correlation was found between the GNRI score and the risk of POD, indicating that malnutrition assessed by the GNRI was a risk factor for POD after cardiac surgery. It should be noted, however, that GRNI cannot reflect the full extent of malnutrition because it neglects some factors, such as vitamin deficiency, sarcopenia, and frailty.^47^ These factors have been demonstrated to be associated with the development of POD.^48^, ^49^, ^50^ The MIMIC database does not contain information regarding these factors, so we did not analyze their association with POD in the present study.

Besides malnutrition, our results also showed that older age, a history of peripheral vascular disease, obstructive sleep apnea, and alcohol abuse, as well as a lower hemoglobin, were independently associated with an increased risk for POD. This result is consistent with previous research.^51^, ^52^ In addition, our results showed that patients who underwent aortic replacement and combined cardiac surgery had a greater likelihood of developing POD (using CABG as a reference). We also showed that patients with POD were more likely to have a higher respiration rate and a higher SOFA score on their first day in the ICU, which suggests that illness severity may contribute to the development of POD. The use of benzodiazepines during ICU stay was also independently associated with a higher risk for delirium. This was consistent with recent evidence and supported the notion that patients at high risk for delirium should reduce or avoid benzodiazepine use.^53^, ^54^

According to our findings, patients at risk of malnutrition are more likely to develop POD. However, due to the observational nature of this study, we were unable to establish a causal relationship between nutritional status and delirium. Nevertheless, there was evidence that certain nutrients may contribute to or cause delirium, such as vitamin D^50^, ^55^ and mega‐3 fatty acids.^56^ Recently, perioperative nutrition intervention has been considered as part of the multidisciplinary approach to preventing delirium.^11^, ^57^, ^58^ However, further well‐designed prospective studies are still needed to determine whether preoperative malnutrition treatment can reduce the incidence of POD.

Our research suggests that the incorporation of preoperative GNRI scores improves the accuracy of POD prediction models. These findings may help to identify patients at risk for delirium at an early stage, so certain preventive interventions can be used to reduce the incidence of POD. It has been reported that infusion of dexmedetomidine can prevent delirium after surgery and in the ICU,^10^, ^59^ although controversy remains.^60^ Numerous non‐pharmacological interventions have also been proposed to prevent delirium.^9^, ^61^ Most of these interventions focus on reversible risk factors, such as sleep promotion, family support, and environmental interventions.^62^, ^63^, ^64^

The present study has the following strengths. First, the cohort size was larger than that of previous studies. Second, we evaluated patients' nutrition status using the GNRI, which is simple, objective, and more suitable for elderly patients. Third, we adjusted for a greater number of confounding factors than in previous studies to determine the association between malnutrition and POD. Nevertheless, some limitations must be acknowledged. First, because the MIMIC database lacks information such as education level, duration of cardiopulmonary bypass during surgery, and perioperative pain scores, we are unable to adjust these confounding factors in the prediction model. Second, the findings of our study were based on a single‐center cohort, which limits their generalizability. Further studies involving multiple centers are required to validate our findings.

In conclusion, our results showed a negative association between the preoperative GNRI score and the risk of POD in elderly patients undergoing cardiac surgery. The addition of GNRI to POD prediction models may improve their predictive accuracy. However, due to the observational nature of this study, we were not able to establish a causal link between nutritional status and POD. In addition, these findings were based on a single‐center cohort and will need to be validated in future studies involving multiple centers. Further research is also required to determine whether preoperative malnutrition evaluation and treatment based on the GNRI could reduce the incidence of POD.

## AUTHOR CONTRIBUTIONS

ZC, HC, and YZ conceived the idea and interpreted the results. RS and QH collected the data. ZC, YZ, and HF extracted the data. ZC, SL, CL, and RS analyzed the data. ZC, QH, and RS drafted the manuscript. HC and YZ revised the manuscript. All authors read and approved the final manuscript.

## CONFLICT OF INTEREST STATEMENT

None.

## Supporting information


Appendix S1.

## Data Availability

The data that support the findings of this study are openly available in the MIMIC IV database (v1.0) at https://physionet.org/content/mimiciv/1.0/.
