# Supplementary material for: Predictive value of the geriatric nutrition risk index for postoperative delirium in elderly patients undergoing cardiac surgery
Source: CNS Neurosci Ther. 2023 Jul 5;30(2):e14343. doi: 10.1111/cns.14343 (PMC10848042; doi:10.1111/cns.14343)
Supplement: Supplementary file 1 — Appendix S1. [file CNS-30-e14343-s001.docx]

# Table S1. ICD-9 and ICD-10 codes for identifying cardiac surgeries and delirium.

|  | ICD-9 codes | ICD-10 codes |
| --- | --- | --- |
| Cardiac surgeries | 3511, 3512, 3514, 3521, 3522, 3523, 3524, 3527, 3611, 3612, 3613, 3614, 3615, 3616, 3619, 3845 | 0210089, 0210093, 0210099, 021009W, 02100A3, 02100A8, 02100A9, 02100AW, 02100Z3, 02100Z8, 02100Z9, 0211093, 0211099, 021109W, 02110A9, 02110AW, 02110Z3, 02110Z8, 02110Z9, 0212093, 0212099, 021209W, 02120AW, 02120Z8, 02120Z9, 0213093, 0213099, 021309W, 02130A3, 02130Z8, 02QF0ZZ, 02QG0ZZ, 02RF08Z, 02RF0JZ, 02RF0KZ, 02RG08Z, 02RG0JZ, 02RG0KZ, 02RJ08Z, 02RW0JZ, 02RX08Z, 02RX0JZ |
| Delirium | 29281, 2930, 2931, 2939, 34831, 34982, 78009, 78097 | F05, G92, G9341, R410, R4182 |

# Table S2 Comparative analysis of preoperative GNRI levels in cardiac surgery patients.

|  | CABG,  n = 2254 | Valve surgery,  n = 1023 | Aortic replacement,  n = 53 | Combined cardiac surgery,  n = 956 | P for overall difference |
| --- | --- | --- | --- | --- | --- |
| Preoperative GNRI | 112.9 (104.7, 120.7) | 114.3 (106.6, 122.5) | 111.8 (103.9, 117.1) | 112.4 (105.1, 120.9) | 0.002 |

GNRI, geriatric nutritional risk index; CABG, coronary artery bypass grafting. Values are expressed as median (interquartile range). P = 0.003 for CABG compared with valve surgery; P = 0.008 for combined cardiac surgery compared with valve surgery.

# Table S3. Characteristics of the included patients (stratified by malnutrition risk).

|  | Without malnutrition risk (GNRI > 98), n = 3894 | With malnutrition risk (GNRI ≤ 98), n = 392 | *P*-value |
| --- | --- | --- | --- |
| Age, years | 74 (69, 79) | 78 (71, 83) | <0.001 |
| Female sex | 1182 (30.4%) | 151 (38.5%) | 0.001 |
| Ethnicity |  |  |  |
| White | 2991 (76.8%) | 296 (75.5%) | 0.605 |
| Others | 903 (23.2%) | 96 (24.5%) |  |
| BMI, kg/m^2^ | 28.4 (25.6, 32.0) | 22.3 (20.4, 24.0) | <0.001 |
| Comorbidity |  |  |  |
| Hypertension | 3255 (83.6%) | 296 (75.5%) | <0.001 |
| Myocardial infarction | 1093 (28.1%) | 154 (39.3%) | <0.001 |
| Congestive heart failure | 1056 (27.1%) | 174 (44.4%) | <0.001 |
| Peripheral vascular disease | 606 (15.6%) | 75 (19.1%) | 0.077 |
| Cerebrovascular disease | 438 (11.2%) | 56 (14.3%) | 0.087 |
| Obstructive sleep apnea | 484 (12.4%) | 10 (2.6%) | <0.001 |
| Diabetes | 1483 (38.1%) | 91 (23.2%) | <0.001 |
| Chronic pulmonary disease | 875 (22.5%) | 105 (26.8%) | 0.061 |
| Chronic liver disease | 118 (3.0%) | 16 (4.1%) | 0.323 |
| Chronic kidney disease | 733 (18.8%) | 108 (27.6%) | <0.001 |
| Alcohol abuse | 91 (2.3%) | 15 (3.8%) | 0.101 |
| Preoperative laboratory findings |  |  |  |
| Albumin, g/dL | 4.1 (3.8, 4.4) | 3.4 (3.2, 3.7) | <0.001 |
| Creatinine, mg/dL | 1.0 (0.8, 1.2) | 1.0 (0.8, 1.3) | 0.834 |
| Hemoglobin, g/dL | 12.8 (11.4, 13.9) | 11.4 (10.1, 12.6) | <0.001 |
| White blood cell count, ×10^9^/L | 7.2 (6.0, 8.6) | 7.1 (5.8, 9.0) | 0.800 |
| Preoperative GNRI | 114.4 (107.5, 122.2) | 93.5 (90.2, 96.1) | <0.001 |
| Type of surgery |  |  | 0.885 |
| CABG | 2040 (52.4%) | 214 (54.6%) |  |
| Valve surgery | 934 (24.0%) | 89 (22.7%) |  |
| Aortic replacement | 49 (1.3%) | 4 (1.0%) |  |
| Combined cardiac surgery | 871 (22.4%) | 85 (21.7%) |  |
| Laboratory findings on the first day in ICU |  |  |  |
| Creatinine, mg/dL | 1.0 (0.8, 1.2) | 1.0 (0.8, 1.3) | 0.074 |
| Hemoglobin, g/dL | 8.8 (7.9, 9.9) | 8.1 (7.5, 9.0) | <0.001 |
| White blood cell count, ×10^9^/L | 15.3 (12.1, 19.2) | 15.1 (11.8, 19.0) | 0.431 |
| Vital signs on the first day in ICU |  |  |  |
| Heart rate, beats/min | 80 (75, 86) | 80 (76, 86) | 0.620 |
| Mean blood pressure, mmHg | 73 (69, 76) | 73 (69, 77) | 0.396 |
| SpO_2_, % | 98 (97, 99) | 98 (97, 99) | <0.001 |
| Respiration rate, breaths/min | 17 (16, 19) | 17 (16, 19) | 0.042 |
| SOFA score on the first day in ICU | 5 (4, 8) | 6 (4, 8) | 0.039 |
| Use of benzodiazepine during ICU stay | 1133 (29.1%) | 125 (31.9%) | 0.272 |
| postoperative delirium | 566 (14.5%) | 93 (23.7%) | <0.001 |
| Length of ICU stay, days | 2 (1, 3) | 2 (1, 4) | <0.001 |
| Length of hospital stay, days | 7 (5, 10) | 10 (7, 14) | <0.001 |
| Hospital mortality | 45 (1.2%) | 11 (2.8%) | 0.012 |

Values are expressed as median (interquartile range) or number of patients (%). BMI, body mass index; GNRI, geriatric nutritional risk index; CABG, coronary artery bypass grafting; ICU, intensive care unit; MAP, mean arterial blood pressure; SpO_2_, percutaneous oxygen saturation; SOFA, Sequential Organ Failure Assessment.

# Table S4. Univariable and multivariable analysis for postoperative delirium (GNRI as a categorical variable).

|  | **Univariable analysis; crude OR (95% CI), *P*-value** | **Multivariable analysis model 1; adjusted OR (95% CI), *P*-value** | **Multivariable analysis model 2; adjusted OR (95% CI), *P*-value** | **Multivariable analysis model 3; adjusted OR (95% CI), *P*-value** | **Multivariable analysis model 4; adjusted OR (95% CI), *P*-value** | **Relative weight (%) in model 4** |
| --- | --- | --- | --- | --- | --- | --- |
| Age, years | 1.06 (1.05-1.08, P < 0.001) | 1.06 (1.05-1.08, P < 0.001) | 1.06 (1.05-1.08, P < 0.001) | 1.06 (1.05-1.08, P < 0.001) | 1.07 (1.05-1.08, P < 0.001) | 18.13 |
| Sex |  |  |  |  |  | 0.17 |
| Male | Ref. | Ref. | Ref. | Ref. | Ref. |  |
| Female | 1.08 (0.90-1.29, P = 0.408) | 0.99 (0.82-1.19, P = 0.903) | 1.04 (0.85-1.26, P = 0.728) | 1.03 (0.84-1.26, P = 0.756) | 0.96 (0.78-1.18, P = 0.705) |  |
| Ethnicity |  |  |  |  |  | 0.24 |
| Others | Ref. | Ref. | Ref. | Ref. | Ref. |  |
| White | 0.95 (0.78-1.15, P = 0.589) | 0.92 (0.75-1.13, P = 0.418) | 0.92 (0.76-1.13, P = 0.446) | 0.90 (0.74-1.11, P = 0.326) | 0.86 (0.70-1.06, P = 0.152) |  |
| Hypertension |  |  |  |  |  | 0.23 |
| No | Ref. | Ref. | Ref. | Ref. | Ref. |  |
| Yes | 1.24 (0.99-1.57, P = 0.072) | 1.08 (0.85-1.38, P = 0.552) | 1.07 (0.84-1.37, P = 0.581) | 1.07 (0.84-1.37, P = 0.606) | 1.03 (0.80-1.33, P = 0.820) |  |
| Myocardial infarction |  |  |  |  |  | 0.65 |
| No | Ref. | Ref. | Ref. | Ref. | Ref. |  |
| Yes | 1.23 (1.03-1.47, P = 0.024) | 1.10 (0.91-1.33, P = 0.333) | 1.07 (0.88-1.30, P = 0.471) | 1.15 (0.94-1.41, P = 0.179) | 1.10 (0.89-1.35, P = 0.387) |  |
| Congestive heart failure |  |  |  |  |  | 3.32 |
| No | Ref. | Ref. | Ref. | Ref. | Ref. |  |
| Yes | 1.87 (1.57-2.22, P < 0.001) | 1.43 (1.19-1.72, P < 0.001) | 1.41 (1.17-1.70, P < 0.001) | 1.36 (1.12-1.65, P = 0.002) | 1.14 (0.93-1.40, P = 0.197) |  |
| Peripheral vascular disease |  |  |  |  |  | 6.00 |
| No | Ref. | Ref. | Ref. | Ref. | Ref. |  |
| Yes | 1.95 (1.59-2.38, P < 0.001) | 1.81 (1.46-2.22, P < 0.001) | 1.81 (1.46-2.23, P < 0.001) | 1.57 (1.26-1.95, P < 0.001) | 1.39 (1.10-1.74, P = 0.005) |  |
| Cerebrovascular disease |  |  |  |  |  | 1.87 |
| No | Ref. | Ref. | Ref. | Ref. | Ref. |  |
| Yes | 1.60 (1.26-2.01, P < 0.001) | 1.30 (1.01-1.66, P = 0.035) | 1.28 (1.00-1.64, P = 0.046) | 1.26 (0.98-1.61, P = 0.062) | 1.25 (0.96-1.60, P = 0.089) |  |
| Obstructive sleep apnea |  |  |  |  |  | 0.80 |
| No | Ref. | Ref. | Ref. | Ref. | Ref. |  |
| Yes | 1.18 (0.92-1.51, P = 0.183) | 1.39 (1.06-1.79, P = 0.014) | 1.39 (1.07-1.80, P = 0.013) | 1.43 (1.09-1.85, P = 0.008) | 1.38 (1.05-1.80, P = 0.020) |  |
| Diabetes |  |  |  |  |  | 0.58 |
| No | Ref. | Ref. | Ref. | Ref. | Ref. |  |
| Yes | 1.15 (0.97-1.36, P = 0.114) | 1.19 (0.99-1.43, P = 0.063) | 1.19 (0.99-1.43, P = 0.070) | 1.24 (1.02-1.49, P = 0.028) | 1.21 (1.00-1.47, P = 0.052) |  |
| Chronic pulmonary disease |  |  |  |  |  | 0.34 |
| No | Ref. | Ref. | Ref. | Ref. | Ref. |  |
| Yes | 1.29 (1.07-1.56, P = 0.008) | 1.08 (0.88-1.31, P = 0.460) | 1.08 (0.89-1.32, P = 0.428) | 1.07 (0.87-1.31, P = 0.518) | 1.02 (0.83-1.25, P = 0.855) |  |
| Chronic liver disease |  |  |  |  |  | 1.94 |
| No | Ref. | Ref. | Ref. | Ref. | Ref. |  |
| Yes | 2.16 (1.45-3.16, P < 0.001) | 1.93 (1.27-2.87, P = 0.002) | 1.95 (1.28-2.90, P = 0.001) | 1.87 (1.23-2.79, P = 0.003) | 1.39 (0.89-2.11, P = 0.137) |  |
| Chronic renal disease |  |  |  |  |  | 1.81 |
| No | Ref. | Ref. | Ref. | Ref. | Ref. |  |
| Yes | 1.77 (1.46-2.13, P < 0.001) | 1.29 (1.04-1.58, P = 0.017) | 1.16 (0.92-1.46, P = 0.217) | 1.16 (0.92-1.47, P = 0.203) | 1.01 (0.79-1.28, P = 0.942) |  |
| Alcohol abuse |  |  |  |  |  | 4.59 |
| No | Ref. | Ref. | Ref. | Ref. | Ref. |  |
| Yes | 2.69 (1.75-4.04, P < 0.001) | 2.78 (1.77-4.28, P < 0.001) | 2.86 (1.82-4.41, P < 0.001) | 2.87 (1.82-4.43, P < 0.001) | 2.47 (1.55-3.87, P < 0.001) |  |
| Malnutrition risk |  |  |  |  |  | 3.76 |
| Without risk (GNRI > 98) | Ref. | Ref. | Ref. | Ref. | Ref. |  |
| With risk (GNRI ≤ 98) | 1.83 (1.42-2.34, P < 0.001) | 1.47 (1.12-1.91, P = 0.005) | 1.51 (1.15-1.98, P = 0.003) | 1.56 (1.18-2.04, P = 0.001) | 1.61 (1.22-2.13, P = 0.001) |  |
| Creatinine before surgery, mg/dL | 1.23 (1.13-1.34, P < 0.001) |  | 1.15 (1.03-1.28, P = 0.009) | 1.15 (1.03-1.28, P = 0.008) | 0.83 (0.62-1.11, P = 0.220) | 1.59 |
| Hemoglobin before surgery, g/dL | 0.93 (0.89-0.97, P = 0.001) |  | 1.03 (0.98-1.09, P = 0.213) | 1.03 (0.98-1.08, P = 0.243) | 1.06 (1.00-1.13, P = 0.047) | 0.45 |
| White blood cell counts before surgery, ×10^9^/L | 1.04 (1.01-1.06, P = 0.006) |  | 1.03 (1.01-1.06, P = 0.017) | 1.03 (1.00-1.06, P = 0.020) | 1.02 (0.99-1.06, P = 0.196) | 1.02 |
| Type of surgery |  |  |  |  |  | 6.40 |
| CABG | Ref. |  |  | Ref. | Ref. |  |
| Valve surgery | 1.04 (0.83-1.29, P = 0.736) |  |  | 1.03 (0.80-1.32, P = 0.817) | 0.96 (0.74-1.25, P = 0.777) |  |
| Aortic replacement | 3.48 (1.91-6.15, P < 0.001) |  |  | 3.13 (1.63-5.87, P < 0.001) | 2.25 (1.12-4.39, P = 0.019) |  |
| Combined cardiac surgery | 1.97 (1.61-2.39, P < 0.001) |  |  | 1.68 (1.35-2.08, P < 0.001) | 1.42 (1.14-1.78, P = 0.002) |  |
| Hemoglobin on the first day in ICU, g/dL | 0.83 (0.78-0.89, P < 0.001) |  |  |  | 0.91 (0.84-0.99, P = 0.026) | 3.05 |
| White blood cell counts on the first day in ICU, ×10^9^/L | 1.01 (1.00-1.03, P = 0.037) |  |  |  | 1.00 (0.98-1.02, P = 0.943) | 0.37 |
| Creatinine on the first day in ICU, mg/dL | 1.29 (1.18-1.41, P < 0.001) |  |  |  | 1.32 (0.98-1.78, P = 0.072) | 2.77 |
| Heart rate on the first day in ICU, beats/min | 1.00 (0.99-1.01, P = 0.442) |  |  |  | 1.00 (0.99-1.01, P = 0.505) | 0.10 |
| Mean MAP on the first day in ICU, mmHg | 0.98 (0.97-1.00, P = 0.009) |  |  |  | 1.01 (0.99-1.02, P = 0.509) | 0.26 |
| Mean SpO_2_ on the first day in ICU, % | 0.98 (0.93-1.05, P = 0.624) |  |  |  | 0.98 (0.92-1.05, P = 0.581) | 0.19 |
| Mean respiration rate on the first day in ICU, breaths/min | 1.06 (1.03-1.10, P < 0.001) |  |  |  | 1.05 (1.01-1.09, P = 0.014) | 2.03 |
| SOFA score on the first day in ICU | 1.18 (1.15-1.22, P < 0.001) |  |  |  | 1.09 (1.06-1.13, P < 0.001) | 16.40 |
| Use of benzodiazepine during ICU stay |  |  |  |  |  | 20.95 |
| No | Ref. |  |  |  | Ref. |  |
| Yes | 2.51 (2.12-2.98, P < 0.001) |  |  |  | 2.15 (1.79-2.60, P < 0.001) |  |

GNRI, geriatric nutritional risk index; CABG, coronary artery bypass grafting; ICU, intensive care unit; MAP, mean arterial blood pressure; SpO_2_, percutaneous oxygen saturation; SOFA, Sequential Organ Failure Assessment; OR, odds ratio; CI, confidential interval.

# Table S5. Univariable and multivariable analysis for postoperative delirium (GNRI as a continuous variable).

|  | **Univariable analysis; crude OR (95% CI), *P*-value** | **Multivariable analysis model 1; adjusted OR (95% CI), *P*-value** | **Multivariable analysis model 2; adjusted OR (95% CI), *P*-value** | **Multivariable analysis model 3; adjusted OR (95% CI), *P*-value** | **Multivariable analysis model 4; adjusted OR (95% CI), *P*-value** |
| --- | --- | --- | --- | --- | --- |
| Age, years | 1.06 (1.05-1.08, P < 0.001) | 1.06 (1.05-1.07, P < 0.001) | 1.06 (1.05-1.08, P < 0.001) | 1.06 (1.04-1.08, P < 0.001) | 1.07 (1.05-1.08, P < 0.001) |
| Sex |  |  |  |  |  |
| Male | Ref. | Ref. | Ref. | Ref. | Ref. |
| Female | 1.08 (0.90-1.29, P = 0.408) | 1.02 (0.84-1.23, P = 0.868) | 1.07 (0.88-1.31, P = 0.503) | 1.07 (0.87-1.31, P = 0.527) | 1.01 (0.82-1.25, P = 0.936) |
| Ethnicity |  |  |  |  |  |
| Others | Ref. | Ref. | Ref. | Ref. | Ref. |
| White | 0.95 (0.78-1.15, P = 0.589) | 0.93 (0.76-1.14, P = 0.459) | 0.93 (0.76-1.14, P = 0.491) | 0.91 (0.74-1.12, P = 0.370) | 0.87 (0.71-1.07, P = 0.190) |
| Hypertension |  |  |  |  |  |
| No | Ref. | Ref. | Ref. | Ref. | Ref. |
| Yes | 1.24 (0.99-1.57, P = 0.072) | 1.07 (0.84-1.37, P = 0.579) | 1.07 (0.84-1.37, P = 0.608) | 1.06 (0.83-1.36, P = 0.637) | 1.03 (0.80-1.33, P = 0.822) |
| Myocardial infarction |  |  |  |  |  |
| No | Ref. | Ref. | Ref. | Ref. | Ref. |
| Yes | 1.23 (1.03-1.47, P = 0.024) | 1.10 (0.90-1.32, P = 0.348) | 1.07 (0.88-1.29, P = 0.499) | 1.15 (0.94-1.40, P = 0.185) | 1.09 (0.88-1.34, P = 0.424) |
| Congestive heart failure |  |  |  |  |  |
| No | Ref. | Ref. | Ref. | Ref. | Ref. |
| Yes | 1.87 (1.57-2.22, P < 0.001) | 1.44 (1.19-1.73, P < 0.001) | 1.42 (1.17-1.71, P < 0.001) | 1.36 (1.12-1.66, P = 0.002) | 1.14 (0.93-1.39, P = 0.212) |
| Peripheral vascular disease |  |  |  |  |  |
| No | Ref. | Ref. | Ref. | Ref. | Ref. |
| Yes | 1.95 (1.59-2.38, P < 0.001) | 1.80 (1.45-2.21, P < 0.001) | 1.80 (1.45-2.21, P < 0.001) | 1.57 (1.25-1.95, P < 0.001) | 1.38 (1.09-1.73, P = 0.006) |
| Cerebrovascular disease |  |  |  |  |  |
| No | Ref. | Ref. | Ref. | Ref. | Ref. |
| Yes | 1.60 (1.26-2.01, P < 0.001) | 1.31 (1.02-1.66, P = 0.032) | 1.29 (1.01-1.64, P = 0.042) | 1.27 (0.99-1.62, P = 0.057) | 1.25 (0.97-1.61, P = 0.081) |
| Obstructive sleep apnea |  |  |  |  |  |
| No | Ref. | Ref. | Ref. | Ref. | Ref. |
| Yes | 1.18 (0.92-1.51, P = 0.183) | 1.44 (1.10-1.87, P = 0.008) | 1.45 (1.11-1.89, P = 0.006) | 1.49 (1.14-1.94, P = 0.004) | 1.46 (1.11-1.92, P = 0.007) |
| Diabetes |  |  |  |  |  |
| No | Ref. | Ref. | Ref. | Ref. | Ref. |
| Yes | 1.15 (0.97-1.36, P = 0.114) | 1.20 (1.00-1.44, P = 0.055) | 1.20 (0.99-1.44, P = 0.057) | 1.25 (1.03-1.51, P = 0.023) | 1.24 (1.02-1.50, P = 0.032) |
| Chronic pulmonary disease |  |  |  |  |  |
| No | Ref. | Ref. | Ref. | Ref. | Ref. |
| Yes | 1.29 (1.07-1.56, P = 0.008) | 1.08 (0.88-1.32, P = 0.439) | 1.09 (0.89-1.33, P = 0.405) | 1.07 (0.87-1.31, P = 0.500) | 1.02 (0.83-1.26, P = 0.827) |
| Chronic liver disease |  |  |  |  |  |
| No | Ref. | Ref. | Ref. | Ref. | Ref. |
| Yes | 2.16 (1.45-3.16, P < 0.001) | 1.93 (1.27-2.87, P = 0.001) | 1.95 (1.29-2.91, P = 0.001) | 1.87 (1.23-2.80, P = 0.003) | 1.37 (0.88-2.10, P = 0.149) |
| Chronic renal disease |  |  |  |  |  |
| No | Ref. | Ref. | Ref. | Ref. | Ref. |
| Yes | 1.77 (1.46-2.13, P < 0.001) | 1.30 (1.05-1.59, P = 0.014) | 1.17 (0.93-1.47, P = 0.183) | 1.18 (0.93-1.48, P = 0.169) | 1.02 (0.80-1.30, P = 0.865) |
| Alcohol abuse |  |  |  |  |  |
| No | Ref. | Ref. | Ref. | Ref. | Ref. |
| Yes | 2.69 (1.75-4.04, P < 0.001) | 2.78 (1.78-4.28, P < 0.001) | 2.86 (1.83-4.41, P < 0.001) | 2.87 (1.83-4.43, P < 0.001) | 2.47 (1.55-3.85, P < 0.001) |
| GNRI | 0.98 (0.98-0.99, P < 0.001) | 0.99 (0.98-1.00, P = 0.023) | 0.99 (0.98-1.00, P = 0.012) | 0.99 (0.98-1.00, P = 0.011) | 0.99 (0.98-0.99, P = 0.001) |
| Creatinine before surgery, mg/dL | 1.23 (1.13-1.34, P < 0.001) |  | 1.15 (1.03-1.27, P = 0.010) | 1.15 (1.03-1.28, P = 0.009) | 0.82 (0.61-1.09, P = 0.187) |
| Hemoglobin before surgery, g/dL | 0.93 (0.89-0.97, P = 0.001) |  | 1.04 (0.98-1.09, P = 0.177) | 1.03 (0.98-1.09, P = 0.207) | 1.07 (1.01-1.13, P = 0.032) |
| White blood cell counts before surgery, ×109/L | 1.04 (1.01-1.06, P = 0.006) |  | 1.03 (1.01-1.06, P = 0.016) | 1.03 (1.01-1.06, P = 0.018) | 1.02 (0.99-1.06, P = 0.187) |
| Type of surgery |  |  |  |  |  |
| CABG | Ref. |  |  | Ref. | Ref. |
| Valve surgery | 1.04 (0.83-1.29, P = 0.736) |  |  | 1.04 (0.81-1.33, P = 0.774) | 0.97 (0.75-1.26, P = 0.843) |
| Aortic replacement | 3.48 (1.91-6.15, P < 0.001) |  |  | 3.04 (1.58-5.69, P = 0.001) | 2.19 (1.09-4.25, P = 0.023) |
| Combined cardiac surgery | 1.97 (1.61-2.39, P < 0.001) |  |  | 1.67 (1.35-2.07, P < 0.001) | 1.43 (1.14-1.79, P = 0.002) |
| Hemoglobin on the first day in ICU, g/dL | 0.83 (0.78-0.89, P < 0.001) |  |  |  | 0.92 (0.84-0.99, P = 0.034) |
| White blood cell counts on the first day in ICU, ×109/L | 1.01 (1.00-1.03, P = 0.037) |  |  |  | 1.00 (0.98-1.02, P = 0.953) |
| Creatinine on the first day in ICU, mg/dL | 1.29 (1.18-1.41, P < 0.001) |  |  |  | 1.34 (0.99-1.82, P = 0.058) |
| Heart rate on the first day in ICU, beats/min | 1.00 (0.99-1.01, P = 0.442) |  |  |  | 1.00 (0.99-1.01, P = 0.516) |
| Mean MAP on the first day in ICU, mmHg | 0.98 (0.97-1.00, P = 0.009) |  |  |  | 1.01 (0.99-1.02, P = 0.501) |
| Mean SpO2 on the first day in ICU, % | 0.98 (0.93-1.05, P = 0.624) |  |  |  | 0.98 (0.91-1.05, P = 0.489) |
| Mean respiration rate on the first day in ICU, breaths/min | 1.06 (1.03-1.10, P < 0.001) |  |  |  | 1.05 (1.01-1.09, P = 0.008) |
| SOFA score on the first day in ICU | 1.18 (1.15-1.22, P < 0.001) |  |  |  | 1.09 (1.06-1.13, P < 0.001) |
| Use of benzodiazepine during ICU stay |  |  |  |  |  |
| No | Ref. |  |  |  | Ref. |
| Yes | 2.51 (2.12-2.98, P < 0.001) |  |  |  | 2.17 (1.80-2.61, P < 0.001) |

GNRI, geriatric nutritional risk index; CABG, coronary artery bypass grafting; ICU, intensive care unit; MAP, mean arterial blood pressure; SpO_2_, percutaneous oxygen saturation; SOFA, Sequential Organ Failure Assessment; OR, odds ratio; CI, confidential interval.
